# Impact of mathematical pharmacology on practice and theory: four case studies

**DOI:** 10.1007/s10928-017-9539-8

**Published:** 2017-09-07

**Authors:** Lambertus A. Peletier, Johan Gabrielsson

**Affiliations:** 1 Mathematical Institute, Leiden University, PB 9512, 2300 RA Leiden, The Netherlands; 2 Division of Pharmacology and Toxicology, Department of Biomedical Sciences and Veterinary Public Health, Swedish University of Agricultural Sciences, Box 7028, 750 07 Uppsala, Sweden

**Keywords:** Receptors, Drug-disposition, Dose-response-time analysis, Michaelis-menten, Quasi-steady-state, Singular perturbations

## Abstract

Drug-discovery has become a complex discipline in which the amount of knowledge about human biology, physiology, and biochemistry have increased. In order to harness this complex body of knowledge mathematics can play a critical role, and has actually already been doing so. We demonstrate through four case studies, taken from previously published data and analyses, what we can gain from mathematical/analytical techniques when nonlinear concentration-time courses have to be transformed into their equilibrium concentration-response (target or complex) relationships and new structures of drug potency have to be deciphered; when pattern recognition needs to be carried out for an unconventional response-time dataset; when what-if? predictions beyond the observational concentration-time range need to be made; or when the behaviour of a semi-mechanistic model needs to be elucidated or challenged. These four examples are typical situations when standard approaches known to the general community of pharmacokineticists prove to be inadequate.

## Introduction

In recent years application of mathematics in drug development has gained momentum. Even the FDA is considering approval of compounds in part on the basis of arguments based on modelling and simulation (cf. [1]). But there is a great variety of ways in which mathematics can play a role in drug discovery and development. On the one hand, the industrial scientist is often faced with the problem to make reliable predictions about such issues as optimal dose or assessment of safety, on the basis of data about onset, intensity and duration of response, when quantitative information about the underlying mechanism of action is limited. The challenge is then to combine available physiological knowledge, well-designed experiments and mathematical analysis to develop a model which can be used to make such reliable predictions. In addition, with expanding knowledge about biological and physiological processes, more systems-based studies are being carried out in which mathematical ideas about dynamical systems are used, for instance, to model complex regulatory networks, or gain insight in the behaviour of such networks, i.e. locate sensitive spots (cf. [2, 3]).

We demonstrate the role mathematics can play in various aspects of pharmacology, such as (i) analysing complex data sets; (ii) using mathematical reasoning for dissecting model structure and acquiring quantitive information out of unconventional response-time courses; (iii) predicting the effect of chronic drug exposure on the basis of relatively short-time data sets in the context of disease progression. Finally, (iv) we show how mathematical analysis can help to discover when a model is not *Well-Posed* 1 [4]. and statistical analysis yields unexpected and counter-intuitive results. We discuss these examples of mathematics in four case studies:

1. *Probing the complexity of Target-Mediated Drug Disposition.*
2. *Using Visual Inspection to estimate model parameters—pattern recognition.*
3. *Model predictions beyond the experimental range.*
4. *Vetting a model that yields counter-intuitive concentration-versus-time graphs.*

The analyses presented in these four cases are based on results published in, respectively [5–8].

## Probing the complexity of target-mediated drug disposition

**Fig. 1:**
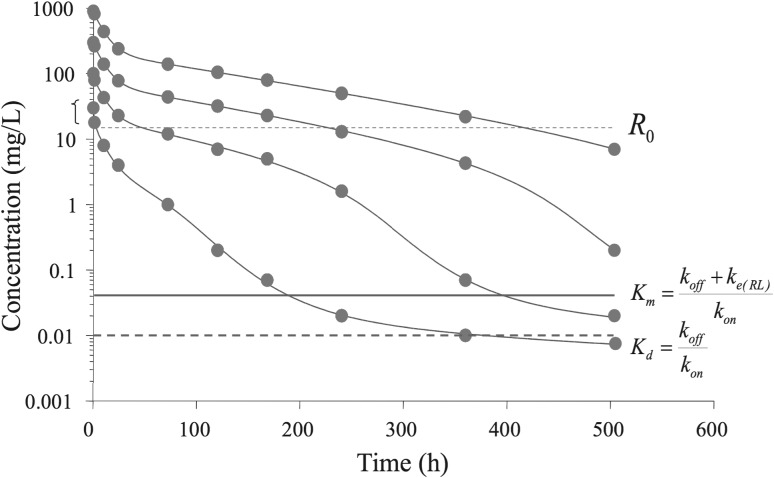
Semi-logarithmic plot of observed (symbols) and TMDD model predicted ligand concentrations in the central compartment (*solid lines*) at four different doses of 1.5, 5, 15 and 45 mg/kg after rapid intravenous injections of a monoclonal antibody. Note that the ligand concentration displays a multi-step pattern that changes in shape as the ligand exposure (dose) decreases. The plot also shows the target baseline concentration in plasma $R_0$, the estimated dissociation constant $K_d$ and the associated Michaelis-Menten constant $K_m$ (cf. Peletier and Gabrielsson [15])

Large molecule compounds, such as monoclonal antibodies, exhibit interesting nontrivial interactions with their target, involving binding, saturation and target turnover. This results in complex ligand-concentration versus time courses. In Fig. 1 we show a typical such data set.

Their dynamics is often referred to as *Target-Mediated Drug Disposition* and has been the subject of many studies (e.g. Mager et al [9–11], Gibiansky et al [12], Krippendorff et al [13], Peletier et al [14, 15], Ma [16] and Dua et al [17]). Characteristically, the concentration-time courses display a series of phases. Initially they exhibit a quick drop, which may easily be missed if the first plasma sample is 12–24 h post dose. Then the curves display a concave bend towards a slower decline. Here, at higher exposure of ligand, one often has first-order linear (dose-proportional) kinetics.

The third typical phase is a convex bend downward with a shorter apparent half-life as we approach lower concentrations. This is where elimination of ligand through target internalisation starts to contribute. The kinetics is now nonlinear. Interestingly, the downward bend occurs at a ligand concentration which is independent of the dose. Throughout this phase the target route of elimination is more or less saturable, but less and less so as concentrations decrease.

Finally, the ligand concentration enters a slower terminal phase, again after a concave bend, with a longer apparent half-life.

### The model

**Fig. 2:**
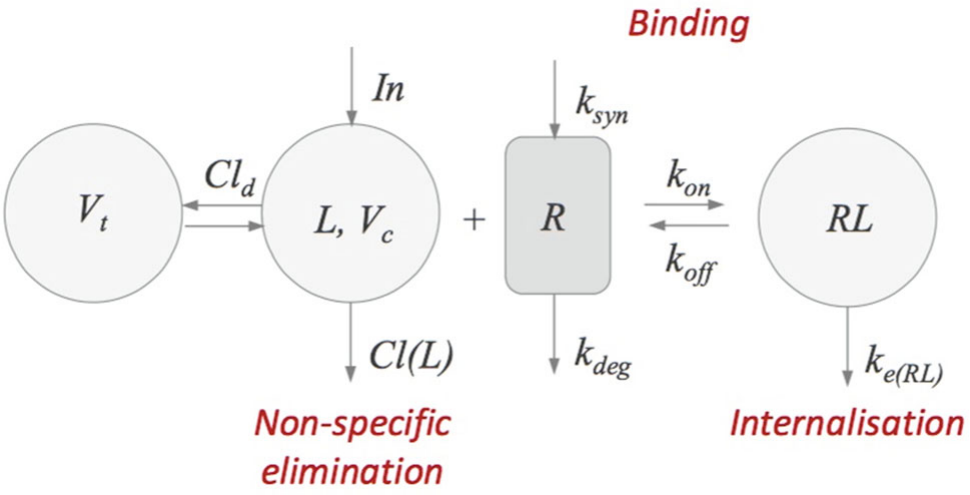
Schematic description of target-mediated drug disposition. Ligand *L* is distributed over a central- and a tissue compartment with respective volumes $V_c$ and $V_t$, is eliminated again via a first order process ($k_{e(L)} = Cl_{(L)}/V_c)$, and binds reversibly ($k_{\mathrm{on}}/k_{\mathrm{off}}$) to the target *R* to form a ligand-target complex *RL*, which then is irreversibly removed via a first order rate process ($k_{e(RL)}$)

The disposition of the antibody (ligand, *L*) is described by a two-compartment disposition model, involving a plasma- and a tissue compartment, coupled to a target pool (*R*) with zero-order production and first-order loss. Ligand and target form a target-ligand complex *RL* via a second-order process. The complex can either be degraded into ligand and target via the first-order $k_{\mathrm{off}}$ process or be irreversibly lost via $k_{e(RL)}$ (first-order internalisation). (Fig. 2)

The combination of the second order formation and first-order loss of complex results in the following nonlinear system involving the concentrations of ligand in the central compartment (*L*) and in the tissue compartment ($L_t$), and of target (*R*) and ligand-target complex (*RL*):

1 $$\begin{aligned} \left\{ \begin{array}{l} \frac{dL}{dt} = k_{\mathrm{infus}} - k_{{e(L)}} \cdot L - k_{\mathrm{on}}L \cdot R + k_{\mathrm{off}} RL- k_{12} L +  k_{21} L_t \\ \frac{dL_t}{dt} = k_{12}L - k_{21}L_t \\ \frac{dR}{dt} = k_{\mathrm{syn}} - k_{\mathrm{deg}} R - k_{\mathrm{on}}L \cdot R + k_{\mathrm{off}} RL\\ \frac{dRL}{dt} = k_{\mathrm{on}} L \cdot R - k_{\mathrm{off}} RL - k_{e(RL)} RL\\ \end{array} \right. \end{aligned}$$

with parameters defined by

2 $$\begin{aligned} k_{\mathrm{infus}}=\frac{Input}{V_c}, \qquad k_{e(L)}=\frac{Cl_L}{V_c}, \qquad k_{12} = \frac{Cl_d}{V_c}, \qquad k_{21} = \frac{Cl_d}{V_t} \end{aligned}$$

in which *Input* denotes the zeroth order input flux, $Cl_L$ the first-order non-specific clearance, $Cl_d$ the inter-compartmental distribution, and $V_c$ and $V_t$ the volume of the central- and the tissue compartment.

### Steady states

**Fig. 3:**
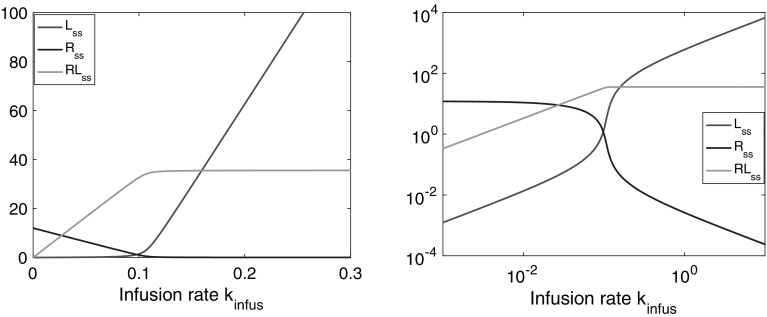
The steady-state concentrations $L_{\mathrm{ss}}$, $R_{\mathrm{ss}}$ and $RL_{\mathrm{ss}}$ graphed versus the infusion rate $k_{\mathrm{infus}}$, on a linear scale (*left*) and on a log-log scale (*right*) for parameter values taken from Table 1

In Fig. 3 we show how the steady-state concentrations of ligand, target and ligand-target complex vary as the infusion rate $k_{\mathrm{infus}}$ changes when the parameter values are given by Table 1. Note that at steady state the ligand concentrations in the central and the peripheral compartment are the same.

**Table 1:** Parameter values taken from Peletier et al, [15]

Parameter	Value	Unit	Description
$k_{e(L)}$	0.0015	h$^{-1}$	Ligand elimination rate
$k_{\mathrm{on}}$	0.091	{(mg/L)h}$^{-1}$	Binding rate
$k_{\mathrm{off}}$	0.001	h$^{-1}$	Dissociation rate
$k_{\mathrm{syn}}$	0.11	(mg/L)/h	Target synthesis rate
$k_{\mathrm{deg}}$	0.0089	h$^{-1}$	Target degeneration rate
$k_{e(RL)}$	0.003	h$^{-1}$	Complex elimination rate
$R_0$	12	mg/L	Target baseline
$R_*$	37	mg/L	Target maximum
$V_c$	0.05	L/kg	Volume central compartment
$V_t$	0.1	L/kg	Volume tissue compartment

For the values of Table 1, the dissociation constant is $K_d=k_{\mathrm{off}}/k_{\mathrm{on}} = 0.011$ mg/L and the Michaelis-Menten constant $K_m=(k_{\mathrm{off}} + k_{e(RL)})/k_{\mathrm{on}}=0.044$ mg/L.

The graphs in Fig. 3 are complex and offer unique diagnostic material to asses the strength of the different processes and the values of the parameters under very general conditions. Besides, whether or not there is a tissue compartment makes no difference, the steady states are the same.

The curves for $L_{\mathrm{ss}}$, $R_{\mathrm{ss}}$ and $RL_{\mathrm{ss}}$, have nontrivial shapes. For instance, in the figure on the left in which they are plotted linearly, they have the following properties:

(i) They each consist of two segments that are approximately linear.
(ii) The two approximately linear segments are joined at a narrow interface located at approximately $k_{\mathrm{infus}}=k_{\mathrm{syn}}=0.11$ (mg/L)/h.

### Mathematical analysis

The slopes of these segments are well-defined and can be computed explicitly. Hence, their dependence on the parameters of the system (1) is transparent. Specifically, for the ligand-receptor complex *RL* one can prove for the slope (*A*) at low infusion rates:

3 $$\begin{aligned} RL_{\mathrm{ss}} \sim A\cdot k_{\mathrm{infus}} \quad {\text {as}} \quad k_{\mathrm{infus}} \rightarrow 0 \qquad {\text {where}}\qquad A \mathop = \limits ^{\mathrm{def}}\frac{1}{k_{e(RL)}}\cdot \frac{R_0}{R_0+\frac{\displaystyle {k_{e(L)}}}{\displaystyle {k_{e(RL)}}}K_m} \end{aligned}$$

At a critical value, when $k_{\mathrm{infus}}\approx k_{\mathrm{syn}}$, the growth of *RL* stops abruptly and the graph becomes flat. The level ($R_*$) at which this happens is given by:

4 $$\begin{aligned} RL_{\mathrm{ss}} \rightarrow R_* \mathop = \limits ^{\mathrm{def}} \frac{k_{\mathrm{syn}}}{k_{e(RL)}} \qquad {\text {as}} \qquad k_{\mathrm{infus}} \rightarrow \infty \end{aligned}$$

Thus, the complex increases more or less linearly up to a plateau where it abruptly levels off. The height of this plateau depends on two parameters only: the synthesis rate of receptor $k_{\mathrm{syn}}$ and the elimination rate $k_{e(RL)}$ of ligand-receptor complex.

Similarly, for the ligand $L_{\mathrm{ss}}$ versus $k_{\mathrm{infus}}$ curve we find for small infusion rates:

5 $$\begin{aligned} L_{\mathrm{ss}} \sim \frac{K_m}{R_0}\cdot A \cdot k_{\mathrm{infus}} \quad {\text {as}} \quad k_{\mathrm{infus}} \rightarrow 0 \end{aligned}$$

For the data of Table 1, $K_m \ll R_0$ so that the initial slope of $L_{\mathrm{ss}}$ is much smaller than that of $RL_{\mathrm{ss}}$. This is also evident in Fig. 3.

For large infusion rates it is possible to prove the following limit

6 $$\begin{aligned} L_{\mathrm{ss}} \sim \frac{1}{k_{e(L)}} (k_{\mathrm{infus}}- k_{\mathrm{syn}}) \qquad {\text {as}} \qquad k_{\mathrm{infus}} \rightarrow \infty \end{aligned}$$

This shows that $L_{\mathrm{ss}}$ climbs as $k_{\mathrm{infus}}$ increases more or less along a straight line with slope $1/k_{e(L)}$ and shifted to the right by an amount equal to $k_{\mathrm{syn}}$.

Because $L_{\mathrm{ss}}$ depends monotonically on the infusion rate, one can also express the steady state values of *RL* and *R* in terms of the steady state ligand concentration. The resulting formula’s are

7 $$\begin{aligned} RL_{\mathrm{ss}}=R_* \frac{L_{\mathrm{ss}}}{L_{\mathrm{ss}}+L_{50}} \qquad {\text {and}} \qquad R_{\mathrm{ss}} = R_0\frac{L_{50}}{L_{\mathrm{ss}}+L_{50}} \end{aligned}$$

in which

8 $$\begin{aligned} R_* = \frac{k_{\mathrm{syn}}}{k_{e(RL)}}= \frac{k_{\mathrm{deg}}}{k_{e(RL)}} \cdot R_0 \qquad {\text {and}} \qquad L_{50} =\frac{k_{\mathrm{deg}}}{k_{e(RL)}}\cdot K_m. \end{aligned}$$

**Fig. 4:**
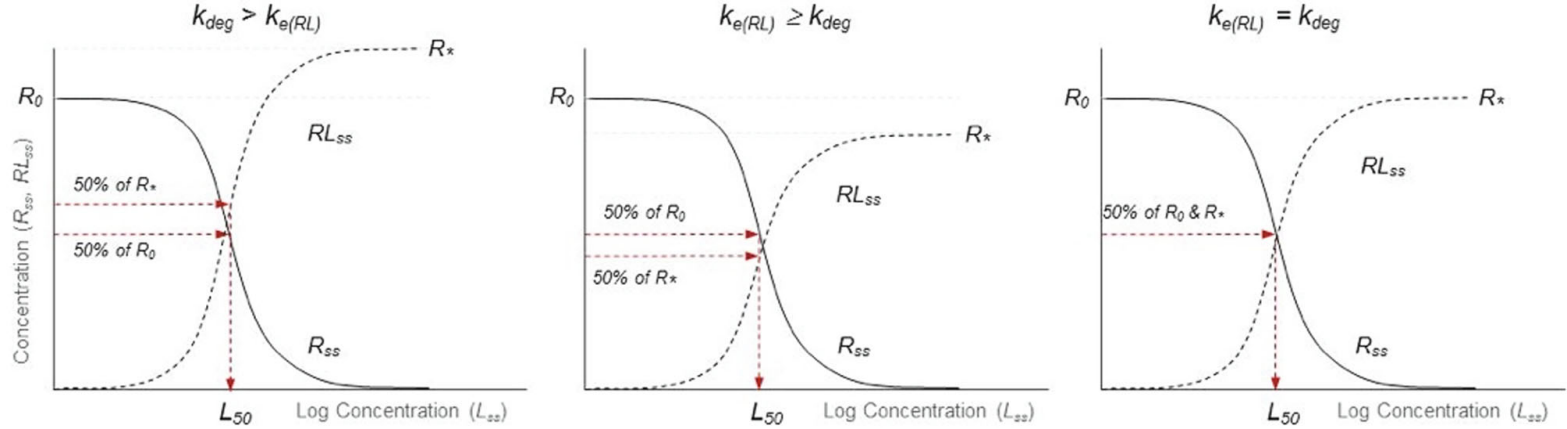
Target suppression $R_{\mathrm{ss}}$ and complex formation $RL_{\mathrm{ss}}$ at equilibrium versus the steady state ligand concentration $L_{\mathrm{ss}}$. The fractional target-turnover rate $k_{\mathrm{deg}}$ is faster (*left*), slower (*middle*) and equal (*right*) than the complex turnover rate $k_{e(RL)}$

According to (7) the graphs of $R_{\mathrm{ss}}$ and $RL_{\mathrm{ss}}$ have a familiar sigmoidal shape with different limits at large and small ligand concentrations. They are shown in Fig. 4:

In particular, the limits at small and large ligand concentrations are given by

9 $$\begin{aligned} \begin{array}{llll} R_{\mathrm{ss}} \rightarrow R_0 \qquad &{}{\text {and}}\qquad &{}RL_{\mathrm{ss}} \rightarrow 0 \qquad &{}{\text {as}}\qquad L_{\mathrm{ss}} \rightarrow 0 \\ R_{\mathrm{ss}} \rightarrow 0 \qquad &{}{\text {and}}\qquad &{}RL_{\mathrm{ss}} \rightarrow R_* \qquad &{}{\text {as}}\qquad L_{\mathrm{ss}} \rightarrow \infty \\ \end{array} \end{aligned}$$

and for both curves the ligand values for which they reach their maximum value is given by

10 $$\begin{aligned} L_{50} =\frac{k_{\mathrm{deg}}}{k_{e(RL)}}\cdot K_m =\frac{k_{\mathrm{deg}}}{k_{e(RL)}}\cdot \frac{k_{\mathrm{off}} + k_{e(RL)}}{k_{\mathrm{on}}} \end{aligned}$$

Detailed information about the asymptotic formulae (3)–(6) and the expressions (7) and (8) can be obtained from explicit analytical expressions for the way the concentrations depend on the infusion rate $k_{\mathrm{infus}}$. They are given in Gabrielsson and Peletier [5].

### Conclusion

We have seen how the complexity of the TMDD model is elegantly depicted by plotting the steady states of the compounds *L*, *R* and *RL* versus the infusion rate $k_{\mathrm{infus}}$ (cf. Fig. 3). The graphs can be computed explicitly and depend critically on the initial amount of target $R_0$ and what one could call a *generalised dissociation constant* which combines dissociation and internalisation of ligand-target complex:

11 $$\begin{aligned} K_m = \frac{k_{\mathrm{off}} + k_{e(RL)}}{k_{\mathrm{on}}} \end{aligned}$$

If $K_m \ll R_0$, a common situation (cf [10, 15, 18]), the graphs are almost piece-wise linear and the explicit expressions for the slopes are quite simple.

Dependence of target-ligand complex formation *RL* and receptor suppression *R* on the ligand concentration, proves to be described by graphs of simple sigmoidal functions (cf. Fig. 4). This introduces a potency parameter $L_{50}$ which is related to $K_m$ by the quotient of the two target elimination rates $k_{\mathrm{deg}}$ and $k_{e(RL)}$:

12 $$\begin{aligned} L_{50} = \frac{k_{\mathrm{deg}}}{k_{e(RL)}}\cdot K_m \end{aligned}$$

Thus, a close analysis of the steady state properties of the three compounds involved in target-mediated disposition reveals a great deal about the relative importance of the sub-processes involved, and offers the possibility to acquire quantitative information about them.

## Using visual inspection to estimate model parameters

This case study is aimed at demonstrating how mathematical reasoning can be used to help the modeller in choosing appropriate models on the basis of pharmacokinetic and pharmacodynamics data sets. This becomes especially important when little is known about the underlying pharmacokinetics, e.g., when the drug is not supplied to the plasma. Thus, for such situations the only data available are for response over time for different doses and forms of administration. Study of such problems is often referred to as *dose-response-time* analysis. It goes back to early papers by Levy [19] and [20] and Smolen [21]. For further references we refer to [6, 22–28]. Specific questions such as (i) *How does mathematical reasoning address what is observed in onset, intensity and duration of response*, and (ii) *How to choose an appropriate model* are discussed.

### Data, model and equations

The data set records the locomotor activity, measured by the number of times moving rats interrupted three light beams in a cage when they were supplied by a drug, (*dexamphetamine*). In the absence of dexamphetamine the number of interruptions was negligible, but it goes up when the drug is given. The exact mode of action of drug is not known, and therefore an empirical mathematical model is proposed.

**Fig. 5:**
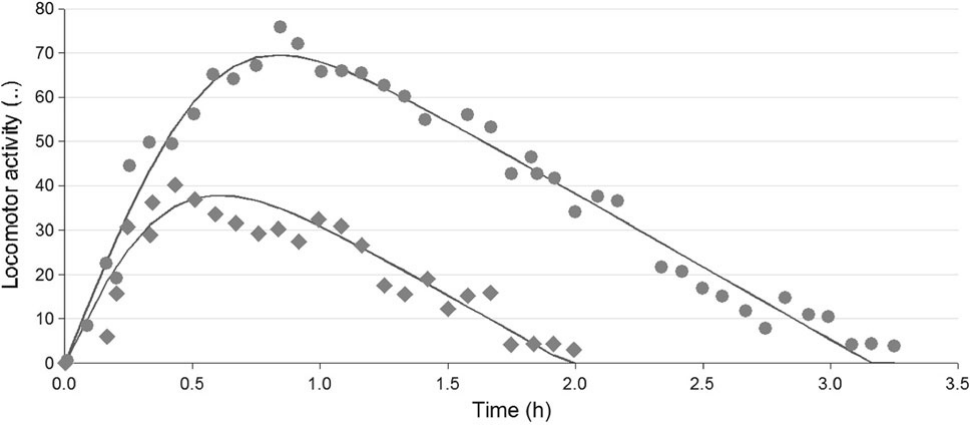
Locomotor activity scores (number of interruptions per minute)-versus-time data following two subcutaneous 3.12 and 5.62 μg kg^−1^ doses of dexamphetamine (Van Rossum and Van Koppen [29]). The *solid lines* represent predictions from fitting the model (14) to the experimental data. Note the apparently linear and parallel decline in response over time independently of dose

Data shown in Fig. 5 were obtained and digitized from Van Rossum and Van Koppen [29]. They recorded the locomotor activity score after administration of dexamphetamine to rats at two dose levels (3.12 and 5.62 μg kg^−1^). The data set was unusual because (i) the rise and drop of response were approximately *linear* with time and (ii) the slope of the increase and of the decline in the locomotor activity score was independent of dose. In addition, the transition from increase to decline was relatively rapid.

Because the exposure to testcompound (drug) was not known, a classic biophase model was fitted, one in which the drug is administered through an intravenous bolus administration. The amount of drug $A_b$ in the biophase (in μg) is described by the equation:

13 $$\begin{aligned} A_b(t) = F^* \cdot D\cdot e^{-k \, t} \end{aligned}$$

where *D* denotes the dose (in μg), and $F^*$ the biophase availability, i.e., the fraction of the dose that reaches the biophase, and *k* the elimination rate of the drug out of the biophase.

**Fig. 6:**
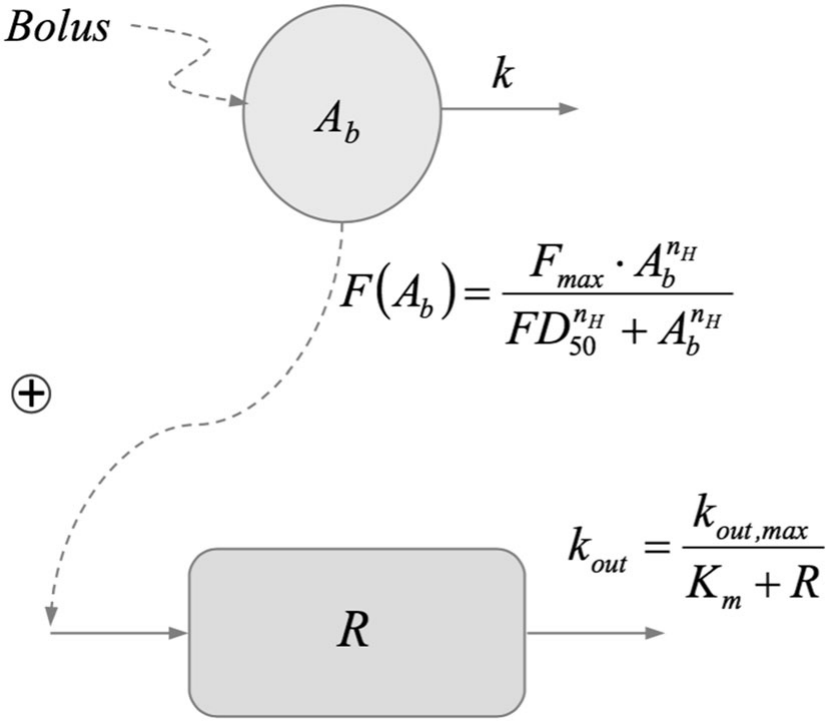
Schematic representation of the locomotor activity model given by eqs. (13) and (14). Drug is supplied to the biophase, eliminated through a first order process (*k*), and the amount of drug $A_b$ has a stimulating effect in the turnover equation for the response through a nonlinear function $F(A_b)$. Loss of response is modeled by a saturable function with maximal (zeroth order) loss rate ($k_{\mathrm{out,max}}$), half of which is reached when $R=K_m$

The pharmacodynamic response *R* i.e., the number of interruptions per minute, is assumed to be described by a *nonlinear* turnover equation

14 $$\begin{aligned} \frac{dR}{dt} = F(A_b) - k_{\mathrm{out,max}} \cdot \frac{R}{K_m + R} \end{aligned}$$

in which $F(A_b)$ is the drug-mechanism function through which the drug in the biophase impacts the response, $k_{\mathrm{out,max}}$ the maximal elimination rate and $K_m$ the response at which the elimination rate is half-maximal. The combined biophase- and pharmacodynamic model is depicted in Fig. 6. Prior to administration of the compound no activity is observed, i.e., $R(0)=0$.

Two observations inform the selection of the function $F(A_b)$:

(i) The data exhibit a zero baseline. This means that $F(0)=0$.
(ii) As the drug dose increases, the initial slope of the data curves appears to reach a maximum.

They suggest a saturable drug-mechanism function which vanishes when the drug vanishes. It is of the following form:

15 $$\begin{aligned} F(A_b) =F_{\mathrm{max}}\frac{A_b^{n_H}}{FD_{50}^{n_H} + A_b^{n_H}} \end{aligned}$$

in which $F_{\mathrm{max}}$ (response units $\cdot t^{-1}$), $FD_{50}$ (dose units) and $n_H$ correspond to the maximum drug-induced efficacy, the potency and the Hill-exponent.

The particular form of the turnover eq. (14) was selected in light of the approximately linear elimination of response which suggest saturation.

### Mathematical analysis

In order to proceed from *qualitative* observations to *quantitative* estimates about the model, we employ the following observations:

- **Decline of the response:** After the time $T_{\mathrm{max}}$ of maximal response the graph has three conspicuous properties: (*i*) it is straight, (*ii*) it does not change with drug dose, and (*iii*) it exhibits a sharp angle as it approaches the baseline.

These characteristics of the response curve offer us an unusual insight into the dynamics of the model.

- At the time of maximal response $T_{\mathrm{max}}$ the drug has been eliminated and $F(A_b)\approx 0$, so that for $t>T_{\mathrm{max}}$, the turnover equation is approximately reduced to
  16 $$\begin{aligned} \frac{dR}{dt} = -k_{\mathrm{out,max}}\frac{R}{K_m+ R} \end{aligned}$$
  When $R \gg K_m$, then eq. (16) simplifies to
  17 $$\begin{aligned} \frac{dR}{dt} = -k_{\mathrm{out,max}} \end{aligned}$$
  which shows that the slope of the graph is $-k_{\mathrm{out,max}}$. On the basis of the data we obtain the estimate:
  18 $$\begin{aligned} k_{\mathrm{out,max}}\approx 29 \quad {\text {interruptions}}\cdot {\text {minute}}^{-1}\cdot \text {h}^{-1} \end{aligned}$$
- The sharp angle of the graph of *R*(*t*) as it approaches the baseline, i.e., when $R\approx 0$, can be accounted for by a small value of $K_m$.
- The response-time course associated with the higher dose peaks at about $T_{\mathrm{max}}=0.8$ h. If one assumes that approximately four half-lives have elapsed before the drug has been cleared from the biophase, this means that $4 \times t_{1/2} \approx 0.8$ h, i.e., the half-life of the drug in the biophase can be approximated by

19 $$\begin{aligned} t_{1/2} \approx 0.2 \quad {\text {h}}, \end{aligned}$$

and

20 $$\begin{aligned} k = \frac{\ln (2)}{t_{1/2}} \approx 3.5 \quad \text {h}^{-1}. \end{aligned}$$

- **Rise of the response** For the higher dose the initial segment of the graph of *R*(*t*) is approximately a straight line. This suggest that during this period the stimulatory function is saturated, so that $F(A_b) \approx F_{\mathrm{max}}$, and, provided $R \gg K_m$, the turnover equation is well approximated by
  21 $$\begin{aligned} \frac{dR}{dt} = F_{\mathrm{max}}-k_{\mathrm{out,max}} \end{aligned}$$
  Since the data show an initial slope (*dR* / *dt*) of 168 interruptions/minute/h, we deduce that $F_{\mathrm{max}}\approx 168+ k_{\mathrm{out,max}}$ interruptions/minute/h. Using the estimate for $k_{\mathrm{out.max}}$ from the first observation, we conclude that
  22 $$\begin{aligned} F_{\mathrm{max}}\approx 168 + 29 = 197\quad {\text {interruptions}}\cdot {\text {minute}}^{-1}\cdot \text {h}^{-1} \end{aligned}$$

#### *Remark*

It is evident from eq. (14) that at *steady state*, the amount $A_{b;{{\text{ss}}}}$ should be small enough so that the production $F(A_{b;{{\text{ss}}}})$ is smaller than the maximal rate of loss in order to reach a steady state. Thus a basic assumption in this model is that

23 $$\begin{aligned} F(A_{b;{{\text{ss}}}})<k_{\mathrm{out,max}} \end{aligned}$$

- **Time to maximal response** $T_{\mathrm{max}}$. To obtain a ball park value for the time to maximal response $T_{\mathrm{max}}$, we approximate the function $F(A_b(t))$, defined by (15), by a step-function. This choice is based on the fact that, as we argued before, the up-swing is more or less linear, so that the function $F(A_b(t))$ appears saturated, and the decline is also linear and in addition, dose-independent, which suggests that $F(A_b(t)) \approx 0$ after the peak-time $T_{\mathrm{max}}$. Thus, if $A_b(t)$ is decreasing and crosses the level $FD_{50}$, say at time *T*, i.e., when $A_b(T)=FD_{50}$, then we postulate that the function $F(A_b(t))$ can be approximated by the step function
  24 $$\begin{aligned} F(A_b(t)) = F_{\mathrm{max}} \cdot {\mathrm{Heav}}(T - t) \end{aligned}$$
  Here ${\mathrm{Heav}}(s)$ denotes the *Heaviside function* which equals +1 if $s \ge 0$ and 0 if $s<0$. Thus, ${\mathrm{Heav}}(T-t)=1$ if $t \le T$ and ${\mathrm{Heav}}(T-t)=0$ if $t>T$.

As the Hill-coefficient becomes larger, this approximation improves, i.e.,

25 $$\begin{aligned} \lim _{n_H \rightarrow \infty } F_{\mathrm{max}}\frac{A_b^{n_H}}{FD_{50}^{n_H}+A_b^{n_H}} = \left\{ \begin{array}{lll} 0 \qquad &{}{\text {if}} \qquad A_b<FD_{50} \\ F_{\mathrm{max}} \qquad &{} {\text {if}} \qquad A_b>FD_{50} \\ \end{array}\right. \end{aligned}$$

This property follows readily from the classical limit

26 $$\begin{aligned} \lim _{n \rightarrow \infty } \frac{x^{n}}{1+x^{n}} = \left\{ \begin{array}{ll} 0 \qquad &{}{\text {if}} \qquad 0<x<1 \\ 1 \qquad &{}{\text {if}} \qquad x>1 \\ \end{array}\right. \end{aligned}$$

The turnover eq. (14) can now be approximated by

27 $$\begin{aligned} \frac{dR}{dt} = F_{\mathrm{max}} \cdot {\mathrm{Heav}}(T-t)-k_{\mathrm{out,max}} \qquad {\text {as}\;\text{long}\;\text{as}}\quad R>0 \end{aligned}$$

except for when *R* is small, specifically, when $R=O(K_m)$.

Starting at baseline, the solution $R^*(t)$ of this equation is given by:

28 $$\begin{aligned} R^*(t) \mathop = \limits ^{\mathrm{def}}  \left\{ \begin{array}{lll} (F_{\mathrm{max}}-k_{\mathrm{out,max}})\, t \qquad &{}{\text {if}} \qquad &{}0 \le t \le T \\ F_{\mathrm{max}}\, T - k_{\mathrm{out,max}}\, t \qquad &{}{\text {if}} \qquad &{}T< t < T_{\mathrm{end}}\\ \end{array}\right. \end{aligned}$$

where $(0,T_{\mathrm{end}})$ is the maximal interval on which $R^*(t)>0$.

Since $F_{\mathrm{max}}>k_{\mathrm{out,max}}$ by eq. (23) it follows that *R*(*t*) is increasing for $0<t<T$. Plainly, *R*(*t*) is decreasing for $t>T$. Therefore $R_{\mathrm{max}} = R(T)$, so that $T=T_{\mathrm{max}}(D)$, and

29 $$\begin{aligned} T_{\mathrm{end}}(D)= \frac{F_{\mathrm{max}}}{k_{\mathrm{out,max}}}\cdot T_{\mathrm{max}}(D) \end{aligned}$$

Because the biophase is assumed to follow intravenous bolus dynamics, as described by eq. (13), it follows that

30 $$\begin{aligned} A_b(T_{\mathrm{max}}) =F^*\cdot D\cdot e^{-k\,T_{\mathrm{max}}}= FD_{50} \end{aligned}$$

We assumed that $F^*=1$, had taken the larger dose $D=5.62$ μg kg^−1^, and estimated the elimination rate out of the system to be $k=3.5$ h$^{-1}$. By visual inspection, the time of maximal response was estimated by $T_{\mathrm{max}}=0.8$. Thus, we conclude from eq. (30) that the potency can be estimated as follows:

31 $$\begin{aligned} FD_{50} = 0.34 \quad \mu {\text {g}}\cdot \mathrm{kg}^{-1}. \end{aligned}$$

Observe that for fixed $A_b$ the steady-state response $R_{\mathrm{ss}}$ is formally given by

32 $$R_{\text{ss}}(A_{b;{{\text{ss}}}}) = K_m \cdot \frac{F(A_{b;{{\text{ss}}}})}{k_{\text{out,max}} - F(A_{b;{{\text{ss}}}})} $$

Recall that according to the assumption (23), we have $k_{\mathrm{out,max}} > F(A_{b;{{\text{ss}}}})$.

### Conclusion

Using mathematical methods we have been able to estimate many of the parameters, such as *k* in the biophase model, and $F_{\mathrm{max}}$ and $FD_{50}$, as well as $k_{\mathrm{out,max}}$ and $K_m$ in the pharmacodynamic model. These estimates can serve as preliminary estimates for further refinement by statistical software. By fitting the model (cf. equation (14)) to the experimental response-time data in Fig. 5, the final parameter estimates of Table 2 were obtained using the nonlinear regression software WinNonlin 5.2 (Certara Inc.).

**Table 2:** Model parameters for the locomotor activity example and relative standard deviation (CV%)

Parameter	Units	Estimate	Final est.	CV%
*k*	h$^{-1}$	3.5	5.96	4
$F_{\mathrm{max}}$	Resp h^−1^	197	249	4
$FD_{50}$	μg kg^−1^	0.34	1.02	4
$n_H$	–	–	1.63	5
$k_{\mathrm{out,max}}$	Resp h^−1^	29	30.1	4
$K_m$	h$^{-1}$	Small	0.001	9

The six model parameters generally had high precision and were close to the analytically and graphically derived initial parameter estimates. The mathematical reasoning in the pattern recognition process thus proves to be useful when unconventional data (as in this case) need to be analysed.

## Model predictions beyond the experimental range

Drug intake over long periods of time, such as is common in the treatment of chronic diseases, may harbour risks which are less evident over the period in which experimental data is available. Simulations on the basis of mathematical models, although predicated by the limited availability of data, may then yield indications of what kind of long time behaviour can be expected and how it can be influenced.

### Data, model and equations

**Fig. 7:**
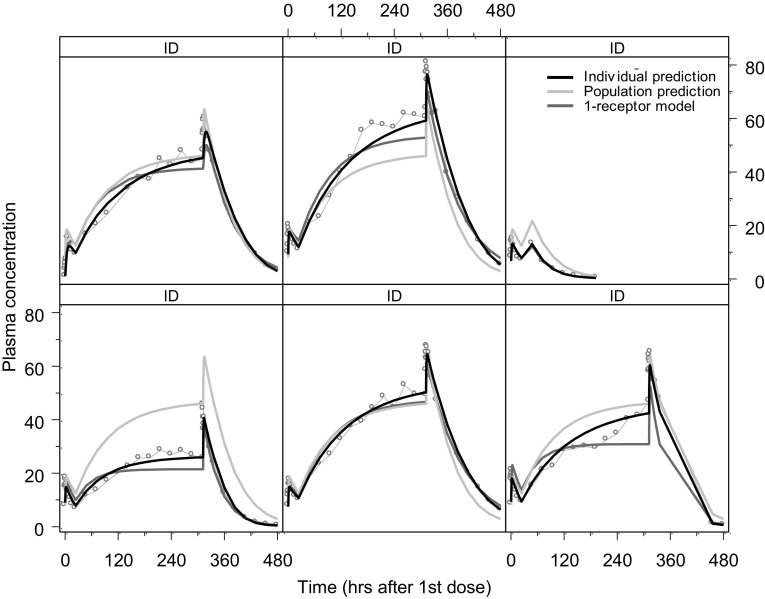
Individual plasma concentration (ng/mL) versus time profiles for six subjects receiving a once-daily oral 1500 mg over a period of 3 weeks. The cyan dots show the observed plasma concentrations, the black curve shows the individual fit. The magenta curves show the individual fits of the 1-receptor model and the grey curve shows the population fit of the 2-receptor model shown in Fig. 8

We consider an example of such a situation discussed by Peletier, de Winter and Vermeulen [7], in which data for the plasma concentration of a compound was available for a period of 480 hours. They are shown in Fig. 7.

**Fig. 8:**
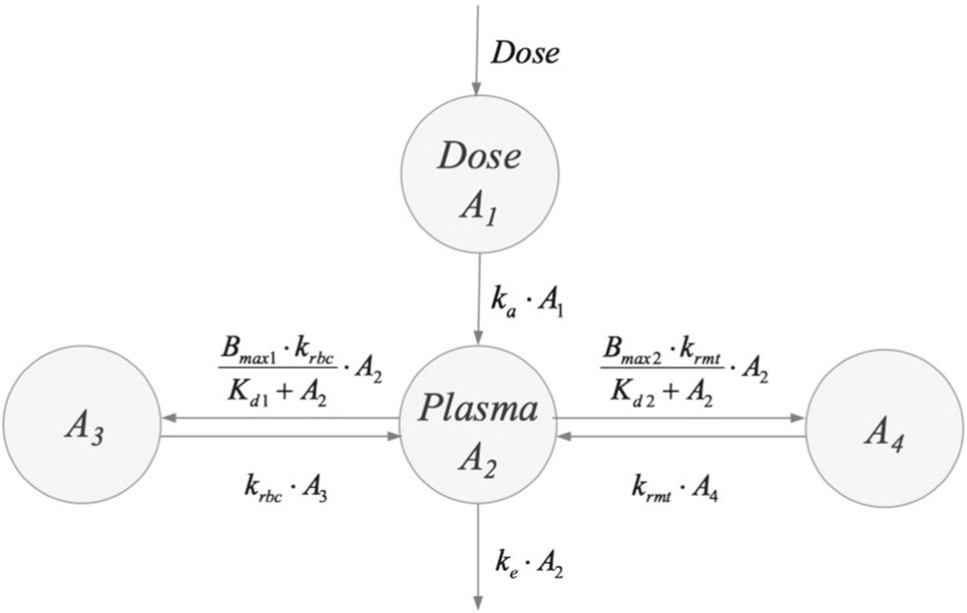
The two-receptor model: the compound is supplied to the pool compartment, from where it reaches the central, plasma, compartment by a first order process ($$k_a $$). From there it is bound by two receptors, one located in red blood cells, with maximal capacity $$B_{{{\text{max}}, 1}}$$ and dissociation constant $$K_{d,1}$$, and the other in a distributed “remote” compartment with maximal capacity $$B_{{{\text{max}}, 2}}$$ and dissociation constant $$K_{d,2}$$, as well as eliminated by a first order process ($$k_e $$) (cf. Snoeck *et al.* [31])

This case-study involves a compound that is administered into a pool compartment and absorbed in a central compartment, from where it binds to two receptors through Michaelis-Menten type reactions (cf. Michaelis and Menten [30]) and dissociates according to first order kinetics. The amounts (in mg) in pool- and central compartment are denoted by, respectively, $A_1$ and $A_2$. Binding to one receptor, which is probably located in the red blood cells is *fast* (amount $A_3$ mg) and binding to the other receptor, which is located in what is called the “remote” compartment, is *slow* (amount $A_4$ mg). The distributional model is illustrated in Fig. 8.

The model used to reach an optimal fit to the data shown in Fig. 7 is based on a 2-receptor model due to Snoeck et al [31] and is shown in Fig. 8. For comparison, the corresponding 1-receptor model is obtained from the above model by putting $k_{\mathrm{RMT}}=0$.

The 2-receptor model for the amounts of compound in the four compartments ($A_1,\dots ,A_4$) translates into the following set of differential equations:

33 $$\begin{aligned} \left\{ \begin{array}{ll} \frac{dA_1}{dt} &{}= D \cdot q - k_a A_1 \\ \frac{dA_2}{dt} &{}= k_a A_1 - k_e A_2- B_{{{\text{max}}, 1}}k_{\mathrm{RBC}} \frac{A_2}{K_{d,1}+A_2} + k_{\mathrm{RBC}} A_3 \\ &{}~~~~~~~~ - B_{{{\text{max}}, 2}}k_{\mathrm{RMT}} \frac{A_2}{K_{d,2}+A_2} + k_{\mathrm{RMT}} A_4\\ \frac{dA_3}{dt} &{}= B_{{{\text{max}}, 1}}k_{\mathrm{RBC}} \frac{A_2}{K_{d,1}+A_2} - k_{\mathrm{RBC}} A_3 \\ \frac{dA_4}{dt} &{}= B_{{{\text{max}}, 2}}k_{\mathrm{RMT}} \frac{A_2}{K_{d,2}+A_2} - k_{\mathrm{RMT}} A_4\\ \end{array}\right. \end{aligned}$$

where $k_{\mathrm{RBC}}$ and $k_{\mathrm{RMT}}$ are the distributional rate constants to the receptors in the red blood cells and the remote receptors, $B_{{{\text{max}}, 1}}$ and $B_{{{\text{max}}, 2}}$ the maximal capacity of these receptors and $K_{d,1}$ and $K_{d,2}$ the associated dissociation constants multiplied by the corresponding volumes. The infusion rate is $D \cdot q$ mg/h, where *q* is the unit infusion rate, i.e. $q=1$ mg/h and *D* is the amount of compound that is supplied per hour.

It is assumed that initially, there is no compound in any of the compartments or bound to the receptors, i.e.,

34 $$\begin{aligned} A_1(0)=0, \qquad A_2(0)=0, \qquad A_3(0)=0, \qquad A_4(0)=0. \end{aligned}$$

From $t=0$ onwards the compound is administered to the pool compartment through a constant-rate infusion of $D\cdot q$ mg/h, which in this study is taken to be 40 mg/h.

Two models are used to fit the data, which are given in aqua. The black curves are the individual fits made with the 2-receptor model (33) and the magenta curves are the individual fits made with the 1-receptor model obtained from the system (33) by putting $k_{\mathrm{RMT}}=0$. The grey curves are the population fits made with the 2-receptor model. In Table 3 we give the parameter values obtained by fitting the 2-receptor model to the data.

**Table 3:** Parameter values

Parameter	Value	Unit	Description
$$k_a $$	2.48	h$$^{-1}$$	Absorption rate
$$k_e $$	0.0111	h$$^{-1}$$	Ligand elimination rate
$k_{\mathrm{RBC}}$	1.06	h$^{-1}$	Rate constant to red blood cells (RBC)
$k_{\mathrm{RMT}}$	0.0000969	h$^{-1}$	Rate constant to remote receptors (RMT)
$B_{{{\text{max}}, 1}}$	77.7	mg	Maximal capacity RBC
$B_{{{\text{max}}, 2}}$	259,000	mg	Maximal capacity RMT
$K_{d,1}$	81.2	mg	Dissociation constant $\times$ $V_c$
$K_{d,2}$	1680	mg	Dissociation constant $\times$ $V_c$
*q*	1	mg/h	Unit infusion rate
*D*	40	–	Dose

Consistent with these parameter values we assume throughout this section that the receptors in the red blood cells have a high affinity to the drug, a small capacity and a short half-time, all relative to the remote receptor. Thus, throughout we assume about the parameter values that:

35 $$\begin{aligned} K_{d,1} \ll K_{d,2}, \qquad B_{{{\text{max}}, 1}} \ll B_{{{\text{max}}, 2}},\qquad k_{\mathrm{RBC}} \gg k_{\mathrm{RMT}} \end{aligned}$$

As drug flows into the system at a rate of *D* mg per hour, the system eventually settles on a *steady state*: ($A_{1,{{\text{ss}}}}, \dots , A_{4,{{\text{ss}}}}$). Plainly, the steady state amounts in the different compartments are given by

36 $$ \begin{array}{ll} {A_{1,{{\text{ss}}}}} = \frac{D}{(k_a/q) }, &\quad {A_{2,{{\text{ss}}}}} = \frac{D}{(k_e/q) }, \\ {A_{3,{{\text{ss}}}}} = {B_{{{\text{max}}, 1}}}\frac{D}{D+(k_e/q) {K_{d,1}}}, &\quad {A_{4,{{\text{ss}}}}} = {B_{{{\text{max}}, 2}}}\frac{D}{D+(k_e/q) {K_{d,2}}} \\ \end{array}$$

Note that $A_{1,{{\text{ss}}}}$ and $A_{2,{{\text{ss}}}}$ are independent of the binding constants and the capacities of the receptors, and increase linearly with the infusion rate *D*.

#### *Remark*

As a preliminary observation we note that the first equation in the system (33) can be solved explicitly, resulting in the following expression for $A_1(t)$:

$$\begin{aligned} A_1(t) = \frac{D\cdot q}{k_a}\,(1-e^{-k_a\,t}) \end{aligned}$$

Thus, $A_1(t)\rightarrow A_{1,{{\text{ss}}}}$ as $t \rightarrow \infty$ with a half-life of $t_{1/2}=\ln (2)/k_a= 0.28$ h, or 17 min. This is exceedingly short for the time scale of interest. So effectively, it is permissible to put $A_1(t) \equiv A_{1,{{\text{ss}}}}$. Therefore, we shall be mainly interested in the amount of drug in the central or plasma compartment and in the two types of receptors.

### Simulations

**Fig. 9:**
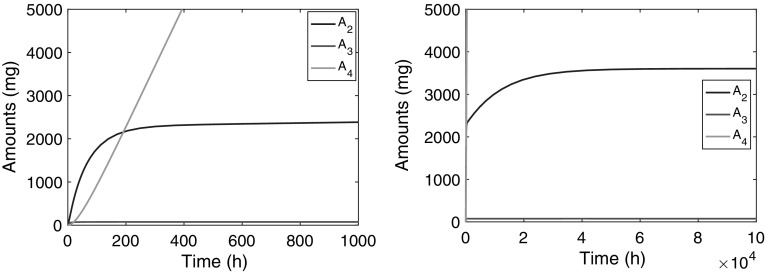
Graphs show the amount versus time courses during a constant-rate infusion over $10^3$ and $10^5$ h of compound in the second compartment ($A_2(t)$ (*blue*)), in the third ($A_3(t)$ (*red*)) and the fourth ($A_4(t)$ (*green*)). The *left panel* shows how over a period of about 500 h $A_2$ climbs to an intermediate steady state and the *right panel* shows how $A_2$ slowly climbs over a much longer period ($5 \times 10^4$ h) to its final steady state $A_{2,{{\text{ss}}}}$. Details about the evolution of $A_3$ and $A_4$ are shown in Fig. 10 (Color figure online)

In Fig. 9 we show how the amount of compound in the plasma compartment, $A_2(t)$, evolves over time after the infusion has been switched on. Evidently, two phases can be distinguished: in the first phase, shown in the left panel, $A_2$ climbs to what appears to be a stationary state, which we usually refer to as the *Plateau value*. Subsequently, during a second phase, shown in the right panel, which extends over a much longer time, $A_2$ continues to climbs towards its final steady state, albeit at a much slower pace.

**Fig. 10:**
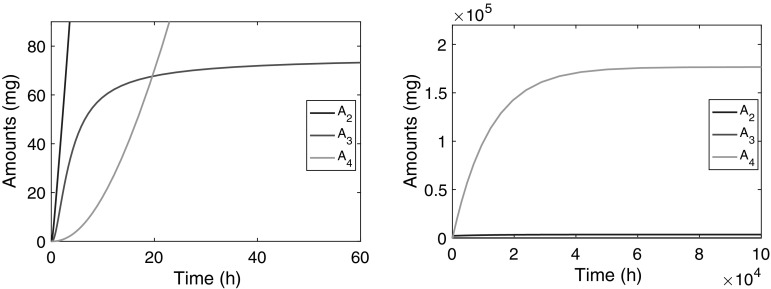
Graphs of $A_2(t)$ (*blue*), $A_3(t)$ (*red*) and $A_4(t)$ (*green*) versus time. The *left panel* shows how over a period of about 50 h $A_3$ climbs to its steady state and in the *right panel* we see how $A_4$ slowly climbs over a much longer period ($5 \times 10^4$ h) to its final steady state $A_{4,{{\text{ss}}}}$ (Color figure online)

In Fig. 10 we show simulations of the amount of compound in the two receptors: $A_3$ in the red blood cells and $A_4$ in remote tissue. The left panel shows that $A_3$ quickly reaches a constant value, which is close to its final steady state $A_{3,{{\text{ss}}}}$ (76.2 mg) computed from (36), the half-life being about 5 h. The right panel shows how $A_4$ evolves over time and reaches its final steady state. Evidently, this takes place on the same time scale as the second phase of $A_2$ shown in Fig. 9.

Summarising we distinguish three time scales in the dynamics of the three-compartment model:

- The receptors in the red blood cells fill up fast. Specifically, $A_3(t)$ reaches its steady-state value with a half-life of $t_{1/2}=O(10)$ h,^2^ which is early compared to the compound in the plasma compartment ($A_2$) and in the remote receptors ($A_4$).
- The central compartment fills up in two phases: fairly quickly up to an intermediate value, the *Plateau Value* $\overline{A}_2$, with a half-life of $t_{1/2}=O(10^2)$ h, and then much more slowly, with a half-life of $t_{1/2}=O(5 \times 10^3)$ h, it creeps up towards its final steady state $A_{2,{{\text{ss}}}}$.
- The remote receptors fill up slowest: the amount of drug $A_4(t)$ reaches its steady-state level with a long half-life $t_{1/2}=O(10^4)$ h.

### Mathematical analysis

In order to understand the observations made about the simulations and answer such questions as (*i*) Which receptor governs the dynamics in the initial phase? (*ii*) How high does $A_2$ rise in the first phase, i.e., what would be a good estimate of the *plateau value* $\overline{A}_2$? (*iii*) What would be the rate of convergence towards $\overline{A}_2$? and (*iv*) What is the rate of convergence towards the final steady state in plasma $A_{2;{{\text{ss}}}}$, and analogous questions about the amount of drug in the two types of receptors.

To answer these questions, we need to compare the relative impact of the different terms in the system (33). Because the amounts in the four compartments $A_1,\dots ,A_4$ vary widely, as, do the rate constants given in Table 4, it is necessary to transform to dimensionless variables and normalise the amounts with well-chosen reference values. In light of the steady state values of $A_2$, $A_3$ and $A_4$ shown in (36), using *D*, $B_{{{\text{max}}, 1}}$ and $B_{{{\text{max}}, 2}}$ as reference values seems appropriate. Thus, we introduce the variables

37 $$\begin{aligned} x(\tau )= \frac{A_2(\tau )}{D}, \qquad y(t)= \frac{A_3(\tau )}{B_{{{\text{max}}, 1}}}, \qquad z(t)= \frac{A_4(\tau )}{B_{{{\text{max}}, 2}}}, \qquad \tau = k_{\mathrm{RBC}}\,t \end{aligned}$$

where we used $1/k_{\mathrm{RBC}}$ as a reference time.

Introducing these variables into the system (33) results in the following system of differential equations:

38 $$\begin{aligned} \left\{ \begin{array}{ll} \frac{dx}{d\tau } &{}= \phi - \mu \, x- \frac{B_{{{\text{max}}, 1}}}{D}\left( \frac{x}{x+\kappa _{1}} -y\right) - \frac{B_{{\text{max}}, 2}}{D}\,\varepsilon \,\left( \frac{x}{x+\kappa _2}-z\right) \\ \frac{dy}{d\tau } &{}= \frac{x}{x+\kappa _{1}} -y \\ \frac{dz}{d\tau } &{}= \varepsilon \,\left( \frac{x}{x+\kappa _{2}} -z\right) \\ \end{array}\right. \end{aligned}$$

where

39 $$\begin{aligned} \phi = \frac{q}{k_{\mathrm{RBC}}}, \quad \mu =\frac{k_e}{k_{\mathrm{RBC}}}, \quad \varepsilon = \frac{k_{\mathrm{RMT}}}{k_{\mathrm{RBC}}}, \quad \kappa _1 = \frac{K_{d,1}}{D} \quad {\text {and}} \quad \kappa _2 = \frac{K_{d,2}}{D} \end{aligned}$$

Note that for the parameter values of Table 4, we obtain $\phi =0.94$, $\mu =0.010$, $\varepsilon =0.0000914$, $\kappa _1 = 2.03$ and $\kappa _2 = 42$, and

40 $$\begin{aligned} \frac{B_{{{\text{max}}, 1}}}{D}= 1.9425 \qquad {\text {and}} \qquad \frac{B_{{{\text{max}}, 2}}}{D}\, \varepsilon =0.59194 \end{aligned}$$

By (34), the initial data are now $x(0)=0$, $y(0)=0$ and $z(0)=0$.

Because $\varepsilon \ll 1$ the right-hand side of the third equation of the system (38) is very small. This means that $z(\tau ) \approx 0$ for times $\tau$ up to order $10^{-1} \times \varepsilon ^{-1}\approx 10^3$. Thus, up till such time we may approximate the full system (38) by putting $z=0$ and replace it by the simpler system which involves only two equations and two unknowns: *x* and *y*:

41 $$\begin{aligned} \left\{ \begin{array}{ll} \frac{dx}{d\tau } &{}= \phi - \mu x- \frac{B_{{{\text{max}}, 1}}}{D}\left( \frac{x}{x+\kappa _{1}} -y\right) - \frac{B_{{{\text{max}}, 2}}}{D}\, \varepsilon \, \frac{x}{x+\kappa _2} \\ \frac{dy}{d\tau } &{}= \frac{x}{x+\kappa _{1}} -y \\ \end{array}\right. \end{aligned}$$

Note that while $\varepsilon$ is very small, when it is multiplied by $B_{{{\text{max}}, 2}}$, as it is in eq. (41), the product is no longer small (cf. eq. (40)).

This system (41) has a unique steady state $(\overline{x},\overline{y})$, where $\overline{x}$ can be computed as the root of the function *f*(*x*) defined below:

42 $$\begin{aligned} f(x) \mathop = \limits ^{\mathrm{def}} \phi - \mu \, x- \frac{B_{{{\text{max}}, 2}}}{D}\, \varepsilon \,\frac{x}{x+\kappa _2} \end{aligned}$$

and $\overline{y}$ can then be computed from the right-hand side of the second equation in (41). Equation (42) is essentially a second order equation in *x* which can be solved explicitly. However, because of the many parameters the expression for $\overline{x}$ is quite messy.

However, an important property of $\overline{x}$ can easily be inferred by inspection: Note that (*i*) the function *f*(*x*) is monotonically decreasing, (*ii*) $f(0)=\phi$ and (*iii*) $f(x_{\mathrm{ss}}) < 0$, where $x_{\mathrm{ss}} = A_{2;{{\text{ss}}}}/ D = \phi /\mu$. Therefore, $0<\overline{x}<x_{\mathrm{ss}}$ and hence $0<\overline{A}< A_{2;{{\text{ss}}}}$, in agreement with the simulations shown in Fig. 9. Note that this conclusion is independent of the values of the parameters. For the parameter values of Table 4, the computed values for $\overline{x}$ and $\overline{y}$ are found to be

43 $$\begin{aligned} \overline{x}=57.4\,\,  {\text  {and}}\,\,\overline{y}=0.965 \qquad (\overline{A}_2 = 2297\;{\text {and}}\;\overline{A}_3 = 75 \,{\text {mg}}) \end{aligned}$$

For small perturbations $(\overline{x}+\xi (\tau ),\overline{y}+\eta (\tau ))$, where $\xi (\tau )$ and $\eta (\tau )$ are small compared to $\overline{x}$ and $\overline{y}$, one can derive a linear system of differential equations for $\xi (\tau )$ and $\eta (t)$ and compute the rate with which orbits converge towards the steady state $(\xi ,\eta )=(0,0)$ as $\tau \rightarrow \infty$, and thus compute the *terminal slope* or *terminal elimination rate* $\lambda _z$, defined by

$$\begin{aligned} \lambda _z \mathop = \limits ^{\mathrm{def}} - \lim _{\tau \rightarrow \infty } \tau ^{-1}\, \ln (\xi (\tau )) \end{aligned}$$

and the half-life by $\tau _{1/2}= \ln (2)/\lambda _z$ in this phase. For the parameter values of Table 4 this amounts to $t_{1/2}=k_{\mathrm{RBC}}^{-1}\cdot \tau _{1/2}=50$ min (cf. [7]). Hence after $4 \times t_{1/2}=200$ h the plateau value has approximately been reached, consistent with the simulations shown in Fig. 9.

Beyond the first phase, $A_2$ and $A_3$ are in quasi-equilibrium and we may assume that

44 $$\begin{aligned} y=\frac{x}{x+\kappa _1} \end{aligned}$$

This enables one to reduce the system (38) to a different, smaller, system making it possible to estimate the half-life of the convergence towards the final steady state $A_{\mathrm{2;ss}}$. In fact, it is found that the terminal slope of this phase is $\lambda = k_{\mathrm{RMT}}$, so that the half-life is given by

45 $$\begin{aligned} t_{1/2} = \frac{\ln (2)}{k_{\mathrm{RMT}}} = 7153\,{\text {h}} \end{aligned}$$

This is also consistent with the findings in Fig. 9 (Note that $t \approx \tau$). For details of the derivation of these estimates we refer to [7].

### Conclusions

The mathematical analysis of the multiple receptor binding system demonstrates that care should be taken when using the model for making long-term predictions since such predictions may involve extended periods which well exceed the duration of experimental data. The final steady state of both binding processes may then be significantly higher than what is reached within the experimental time span. Therefore, long term exposure data will be needed to validate the model if used for future risk assessment.

The insights obtained from this mathematical analysis will support the development of alternative models that exhibit the same short to medium term kinetics, but different long term kinetics provided chronic exposure data are available for model validation. For example, they could quantify the impact of small leakage, which over extended periods, may well be large (cf. [32]).

## Vetting a model that yields counter-intuitive concentration-versus-time graphs

In pharmacology, mechanistic mathematical models are commonly developed on the basis of a combination of what is known about the underlying physiology and statistical methods which attempt to estimate the parameters in the model using experimental data. The resulting model is then employed to make predictions about optimal drug dose and generally, about the temporal behaviour of the drug and its effect. In general, before using the model in a clinical environment, it is “challenged” against different drug doses for which data sets exist, or for completely different data sets. In some cases this yields unexpected results. To get to the bottom of such an apparent anomaly a mathematical study of the model is then advised. In this case study we study a recent mechanistic model for Prolactin (PRL) which yielded unexpected results about the dynamics of prolactin (cf. [8] and [33]) when fitted to pharmacokinetic data from Kozielska et al [34].

### The prolactin model

This case study involves a model designed to investigate the response of PRL to antipsychotic drugs, such as *remoxipride* or *risperidone*, in rats. The model, developed by Movin-Osswald et al., [35], which is based on the classical precursor-pool model [36–38] which distinguishes between PRL in plasma, and PRL in lactotrophs that serves as a precursor pool for the PRL in plasma. If *P* denotes the PRL concentration in the lactotrophs and *R* the PRL concentration in plasma, then this model is given by the following system of equations:

46 $$\begin{aligned} \left\{ \begin{array}{ll} \frac{dP}{dt} &{}= k_{\mathrm{s}} - k_{\mathrm{r}}\{1+S(C)\}\,P \\ \frac{dR}{dt} &{}= k_{\mathrm{r}}\{1+S(C)\}\,P - k_{\mathrm{el}}\,R\\ \end{array}\right. \end{aligned}$$

Here $k_{\mathrm{s}}$ denotes the zeroth order synthesis rate of PRL, $k_{\mathrm{r}}$ the first order rate of release of PRL from the lactotrophs into plasma, and $k_{\mathrm{el}}$ the first order elimination rate of PRL from plasma. The drug, at concentration *C*(*t*) in the brain, stimulates the release rate from lactotrophs through a standard saturable drug-mechanism function *S*(*C*) defined by

47 $$\begin{aligned} S(C) = S_{\mathrm{max}}\frac{C}{SC_{50}+C} \end{aligned}$$

where $S_{\mathrm{max}}$ is the maximal extent of stimulation, $SC_{50}$ the drug dose for which stimulation reaches 50% of its maximal effect, and $n_ H$ the Hill exponent.

Evidently, the baseline $(P_0,R_0)$ is given by

48 $$\begin{aligned} P_0 = \frac{k_{\mathrm{s}}}{k_{\mathrm{r}}} \qquad {\text {and}} \qquad R_0 = \frac{k_{\mathrm{s}}}{k_{\mathrm{el}}} \end{aligned}$$

**Fig. 11:**
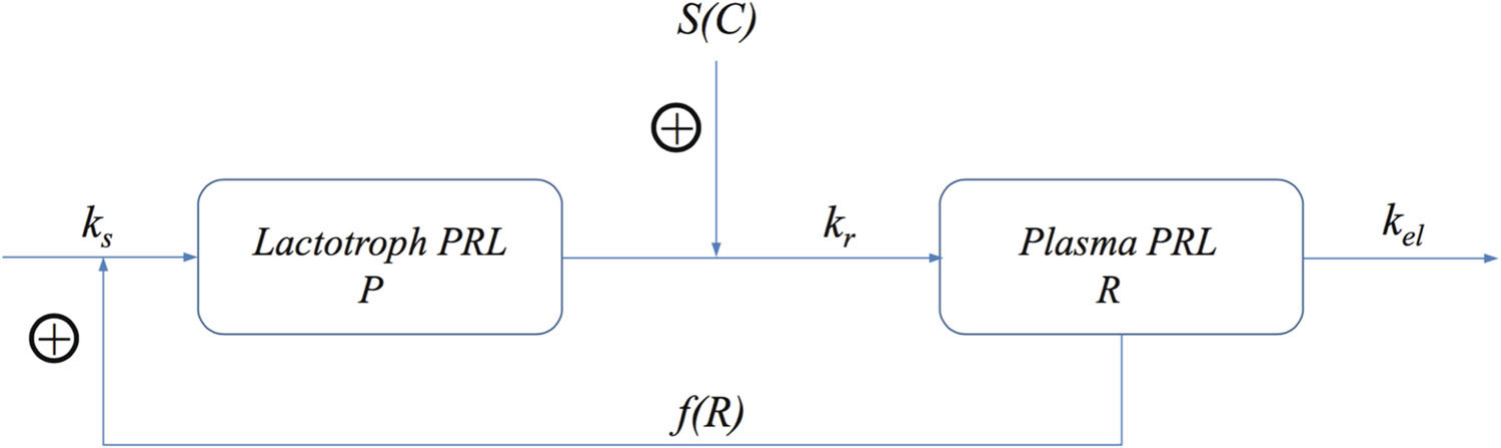
Lactotrophs-Prolactin pool model with positive feedback *f*(*R*)

Stevens et. al., [33] incorporated the fact that release of prolactin by the lactotrophs into plasma has a stimulating effect on the production of prolactin resulting in a *positive feedback loop* (see Fig. 11). See also Friberg et. al., [39].

They incorporated that effect into the model (46) through a multiplicative function of the synthesis rate $k_{\mathrm{s}}$ that depends on the prolactin concentration in plasma, generalising the classical model (46) to the following model

49 $$\begin{aligned} \left\{ \begin{array}{ll} \frac{dP}{dt} &{}= k_{\mathrm{s}}\{1+f(R)\} - k_{\mathrm{r}}\{1+S(C)\}\,P \\ \frac{dR}{dt} &{}= k_{\mathrm{r}}\{1+S(C)\}\,P - k_{\mathrm{el}}\,R\\ \end{array}\right. \end{aligned}$$

In this model *f*(*R*) is a non-decreasing function of *R* endowed with the following properties: (i) For $R \ge R_0$, the function *f*(*R*) is strictly increasing which vanishes when prolactin is at baseline, i.e., when $R=R_0$. (ii) To guard against sudden collapse, the feedback is switched off when the PRL concentration drops below the baseline value, i.e., $f(R)=0$ when $R < R_0$. Stevens et al [33] chose a function of the following form:

50 $$\begin{aligned} f(R) = f_0(R) \cdot {H}(R-R_0), \qquad f_0(R) = \frac{E_{\mathrm{max}} (R-R_0)}{EC_{50}+(R-R_0)} \end{aligned}$$

in which $E_{\mathrm{max}}$ denotes the maximal stimulatory effect on the synthesis rate $k_{\mathrm{s}}$ and $EC_{50}$ the increase of the PRL concentration above the baseline value at which half the maximal effect is achieved. In addition, $H(R-R_0)$ is the *Heaviside function* which vanishes for $R\le R_0$ and so ensures that $f(R)=0$ for $R < R_0$, and equals to 1 for $R \ge R_0$ so that $f(R)=f_0(R)$ for $R \ge R_0$.

### Counterintuitive behaviour

**Fig. 12:**
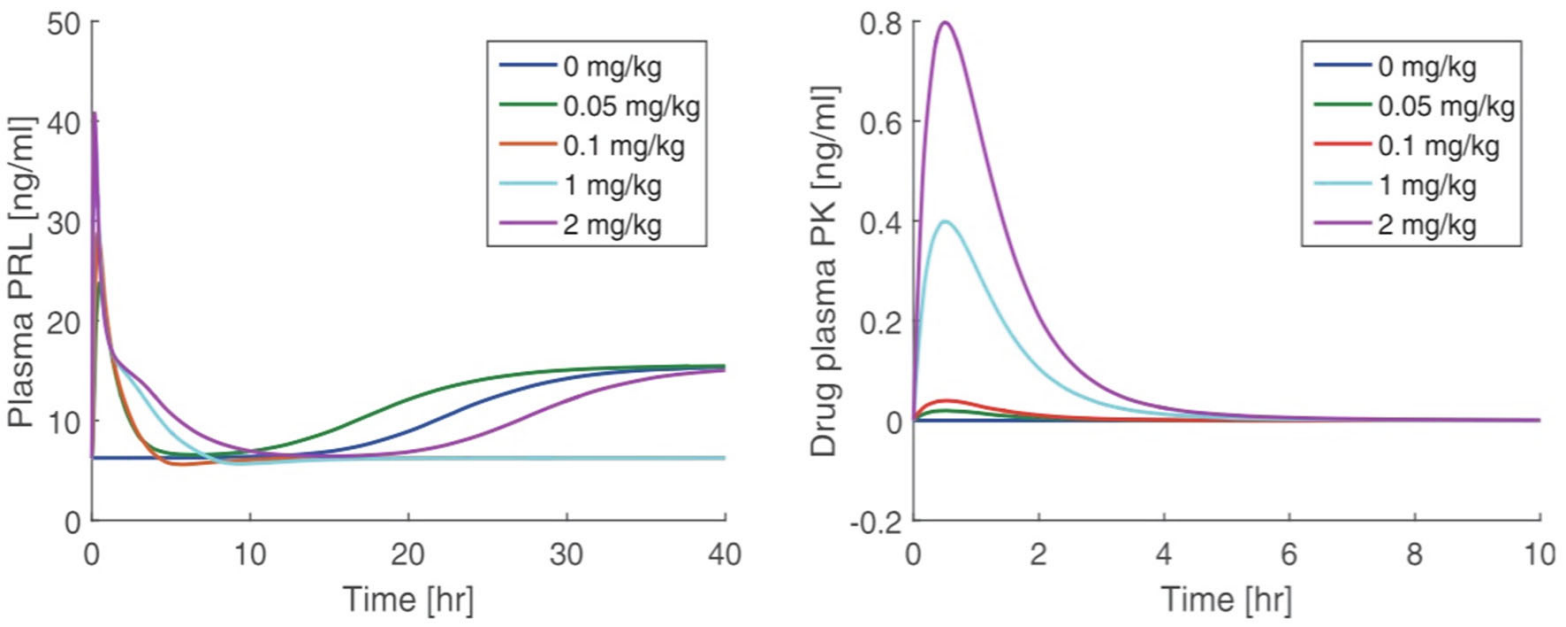
Counterintuitive dynamics seen in PRL concentration in plasma (on the *left*): for $D=0.1$ and 1 mg/kg the PRL-concentration returns to baseline, whilst for $D=0.05$ and 0.2 mg/kg it converges to a higher constant state. The *right* figure shows graphs of the drug concentration versus time corresponding to the four doses based on the model by Kozielska et al [34]

When Proost and Taneja (Private communication) used PK data for risperidone obtained by Kozielska et al [34] (cf. Appendix) to drive the model above, they found the following counterintuitive behaviour (cf. Fig. 12):

1. For drug doses $D=0.1$ and 1.0 mg/kg they observed that simulations return *R* to the baseline $R_0$ as $t \rightarrow \infty$.
2. For drug doses $D=0.05$ and 2.0 mg/kg they observed that simulations do not return *R* to the baseline $R_0$ as $t \rightarrow \infty$, but converge towards a higher constant level, which we denote by $R_1$.
3. The PK is fast, in that the half-life of drug in the central compartment is around 1 h, whilst the time for *R* to reach $R_0$ is about 10 h and to reach $R_1$ is around 40 h.

This suggests that, (i) when $C(t) \equiv 0$, there exists besides the baseline $R_0$ an additional steady state $R_1>R_0$, and (ii) the drug dependence of the dynamics is not monotone and quite sensitive to drug dose. For practical situations this is very critical so that it is important to find the reasons for this behaviour.

In order to understand these phenomena it is necessary to study the mathematical properties of the model (49) with positive feedback function (50) more closely. That will be done in the next subsection.

### Mathematical analysis

To simplify the equations and reduce the number of parameters, we introduce dimensionless variables. To this end we scale *P* and *R* by their respective baseline values and time by $1/k_{el}$ and put:

51 $$\begin{aligned} x=\frac{P}{P_0}, \qquad ~~ y=\frac{R}{R_0}, \qquad ~ \tau = k_{\mathrm{el}}\,t \end{aligned}$$

Introducing these variables into the system (49) and the feedback function *f*(*R*) we obtain

52 $$\begin{aligned} \left\{ \begin{array}{ll} \frac{dx}{d\tau } &{} = \alpha \left\{ 1+\varphi (y)-\psi (\tau )\,x \right\} \\ \frac{dy}{d\tau } &{} = \psi (\tau )\,x - y \\ \end{array}\right. \qquad \qquad \alpha =\frac{k_{\mathrm{r}}}{k_{\mathrm{el}}} \end{aligned}$$

where $$\psi (\tau ) = 1+S(C(t))$$ and

53 $$\begin{aligned} \varphi (y)=\frac{\beta (y-1)}{\gamma + (y-1)}\cdot H(y-1), \qquad \qquad \beta = E_{\mathrm{max}}, \qquad \gamma = \frac{EC_{50}}{R_0} \end{aligned}$$

### Stationary solutions

Suppose that the drug concentration is constant, i.e. $C(t) \equiv \overline{C}\ge 0$, and $\psi (\tau ) \equiv \overline{\psi }= S(\overline{C})$. Then, by (52) a stationary solution $(\overline{x},\overline{y})$ satisfies the pair of equations

$$\begin{aligned} 1+\varphi (\overline{y})= \overline{\psi }\cdot \overline{x}\qquad {\text {and}} \qquad \overline{\psi }\cdot \overline{x}= \overline{y}\end{aligned}$$

Substituting the second equation into the first yields the following equation for $\overline{y}$:

54 $$\begin{aligned} 1+\frac{\beta (\overline{y}-1)}{\gamma + (\overline{y}-1)} =\overline{y}\end{aligned}$$

This is a quadratic equation in $\overline{y}$ which has the roots:

55 $$\begin{aligned} y_0=1 \qquad {\text {and}} \qquad y_1=1+\beta -\gamma \end{aligned}$$

Plainly, $y_0$ corresponds to $R_0$, the baseline in the absence of positive feedback. However $y_1$ corresponds to a new stationary solution which is introduced by the positive feedback, denoted by $R_1$.

It is illustrative to follow the dynamics of the system in what one may refer to as the *state space*, the (*x*, *y*)-plane in which the state, defined by *x* and *y*, travels. It is often called the *Phase plane* and the trajectory, traced by the concentration pair (*x*(*t*), *y*(*t*)), is called the *Orbit*. Plainly, at each point (*x*, *y*) in this plane the *velocity vector* ${\mathbf{q}}=(dx/d\tau ,dy/d\tau )$ can be computed from the system (52). States at which *x* increases with time $(dx/d\tau >0)$ and where *x* decreases with time $(dx/d\tau <0)$ are separated by curves $\Gamma _x$ where $dx/d\tau =0$. Similarly, $\Gamma _y$ separates states where *y* increases, respectively decreases. The curves $\Gamma _x$ and $\Gamma _y$ are called the *Null clines*. Clearly, the stationary states are located at the points where $\Gamma _x$ and $\Gamma _y$ intersect.

When $\overline{C}=0$, then by the system (52), the null clines are given by

56 $$\begin{aligned} \Gamma _x: ~~y = x \qquad {\text {and}} \qquad \Gamma _y:~~ y = 1+\frac{\gamma (x-1)}{\beta - (x-1)}, \qquad (x \not = \beta +1) \end{aligned}$$

**Fig. 13:**
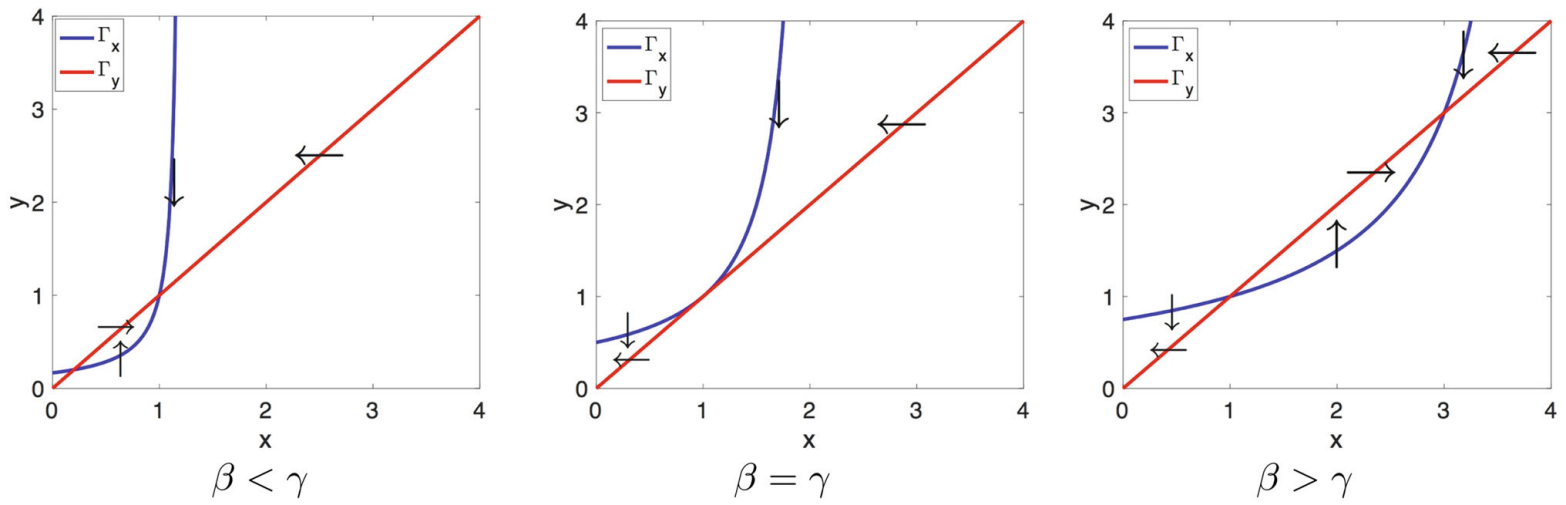
Null clines in the phase plane spanned by the dimensionless concentrations of PRL in lactographs (*x*) and in plasma (*y*) for three ranges of $\beta$ with respect to $\gamma$ (Here $\gamma =1$ and $\beta =0.2,\, 1, \,3$). The *arrows* indicate the direction of the velocity field **q**: *vertical* on $\Gamma _x$ and *horizontal*on $\Gamma _y$

These null clines are shown in Fig. 13 for $\gamma =1$ and $\beta = 0.2,~1.0$ and 3. Notice that $\Gamma _y$ is fixed and $\Gamma _x$ moves to the right and up as $\beta$ increases. As predicted by eq. (55), we see that the corresponding steady states are, besides $(x_0,y_0) = (1,1)$: $(x_1,y_1) =(0.2,0.2)$ when $\beta =0.2$, and $(x_1,y_1) =(3,3)$ when $\beta =3$.

The null clines are very helpful in determining various aspects of the dynamics of the system, such as (i) the stability of the steady states, (ii) the large-time behaviour of orbits: e.g. where they go to and how they approach the stable steady states and (iii) identification of invariant regions, i.e., regions in the plane which trap orbits. Thus, the arrows in Fig. 13 suggest that when the positive feedback is small, i.e., when $\beta = E_{\mathrm{max}}$ is small, then $(x_0,y_0)=(1,1)$ is stable because all the arrows point towards it. However, when the positive feedback becomes stronger, and specifically, when $\beta$ becomes larger than $\gamma$, then $(x_0,y_0)$ loses its stability and arrows point to $(x_1,y_1)$.

When the parameters in the system explicitly depend on time, the situation is more complex. In the system (52) the parameters are all constants except the coefficient $\psi (\tau )$ which depends on $\tau$. However, since $C(t) \rightarrow 0$ very quickly (cf. Fig. 12, right panel), for most of the orbit we may put $C=0$, and hence $\overline{\psi }=1$, after a brief initial period.

The specific parameter values employed for the system (52) by Stevens et al [33] are given in Table 4.

**Table 4:** Parameter values used in [33]

Parameter	Value	Unit	Description
$k_{\mathrm{s}}$	35.7	ng mL^−1^ h^−1^	Synthesis rate PRL
$k_{\mathrm{r}}$	0.59	h$^{-1}$	Release rate PRL
$k_{\mathrm{el}}$	5.7	h$^{-1}$	Elimination rate PRL
$S_{\mathrm{max}}$	25	–	Maximal stimulation
$SC_{50}$	0.08	ng mL^−1^ h	Drug concentration when stimulation is half-maximal
$E_{\mathrm{max}}$	3.47	–	Maximal positive feedback
$EC_{50}$	12.4	ng mL^−1^ h	Value of $R-R_0$ when feedback is half-maximal

They yield for the dimensionless constants: $\alpha =0.10$, $\beta =3.47$ and $\gamma =1.99$. Thus, for the data used in [33] we conclude that $\beta >\gamma$, so that the right-hand graph in Fig. 13 applies. For the baseline we obtain $R_0 = 6.24$ ng mL^−1^ and, using eq. (55), we obtain for the upper steady state $R_1=R_0 \times y_1 = R_0(1+\beta -\gamma )=15.48$ ng mL^−1^, in agreement with the simulation shown in Fig. 12.

Summarising and rephrasing the observations made in the simulations shown in Fig. 12 we can state that

57 $$ \begin{aligned} \left\{ \begin{array}{l} R(t) \rightarrow R_0 \quad {\text {as}}\quad t \rightarrow \infty \qquad {\text {when}} \qquad D=0.1 \& 1.0\quad {\text {mg/kg}}, \\ R(t) \rightarrow R_1 \quad {\text {as}} \quad t \rightarrow \infty \qquad {\text {when}} \qquad D=0.05 \& 2.0\quad {\text {mg/kg}} \\ \end{array}\right. \end{aligned}$$

and the question is, why the PRL concentration does not go back to the baseline $R_0$ for any initial dose. In the next subsection we attempt to shed light on this observation.

### Behaviour explained

In order to understand why the behaviour of the PRL-concentration is so sensitive to the drug dose, we turn to the phase plane and follow the orbit traced by the concentration pair (*x*, *y*) from its starting point $(x,y)=(1,1)$ all the way towards its limiting state $(x_\infty ,y_\infty )$. Specifically, we wish to know when the orbit tends to $(x_0,y_0)$ and when to $(x_1,y_1)$, and how the drug dose *D* enters into this selection.

**Fig. 14:**
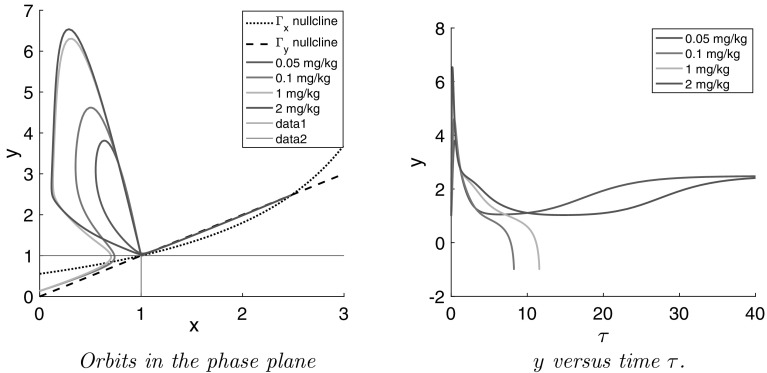
Simulations of the positive feedback model *without cut-off* in the phase plane spanned by the dimensionless concentrations of PRL in lactographs (*x*) and in plasma (*y*) (*Left*) and dimensionless PRL in plasma versus dimensionless time ($\tau$) (*Right*) The doses *D* of risperidone are: *D* = 0.05, 0.1, 1.0 and 2.0 mg/kg. The drug PK is simulated using the PK model for *iv* dose of risperidone [34] (cf. Appendix A )

For simplicity we first study the dynamics of the system (52) in the absence of a cut-off of the positive feedback. For this case we show in Fig. 14 the orbits in the phase plane for the drug doses $D=0.05$, 0.1, 1.0 and 2.0 mg/kg.

We observe in Fig. 14 that after a rapid introduction of risperidone, all orbits leave the baseline $(x_0,y_0)=(1,1)$ move up and to the left along an orbit which is initially tangent to the line

58 $$\begin{aligned} \ell : \quad y=1+\frac{1}{\alpha }(1-x) = 1+10 \times (1-x). \end{aligned}$$

After describing a big loop the orbits all return to a neighbourhood of the baseline $(x_0,y_0)=(1,1)$ from where they started. However, because $\beta >\gamma$ the baseline is unstable and orbits move away from it, with the exception of two orbits: one from above and one from below, which tend towards $(x_0,y_0)$. Orbits which pass above these “stable orbits” (cf. $D=0.05$ and 2 mg/kg) ultimately converge towards the second equilibrium solution $(x_1,y_1)$. Those which pass below (cf. $D=0.1$ and 1 mg/kg) leave the first quadrant and they do this through the *y*-axis, *below the line* $y=1$. This dichotomy is clearly demonstrated in Fig. 14.

Evidently, orbits entering the neighbourhood of $(x_0,y_0)$ are very sensitive to small changes in paramater values or to the drug dose: a small change may flip them to the other side of the two stable orbits and change their subsequent course dramatically. This is the phenomenon that Proost and Taneja observed.

### Including cut-off

**Fig. 15:**
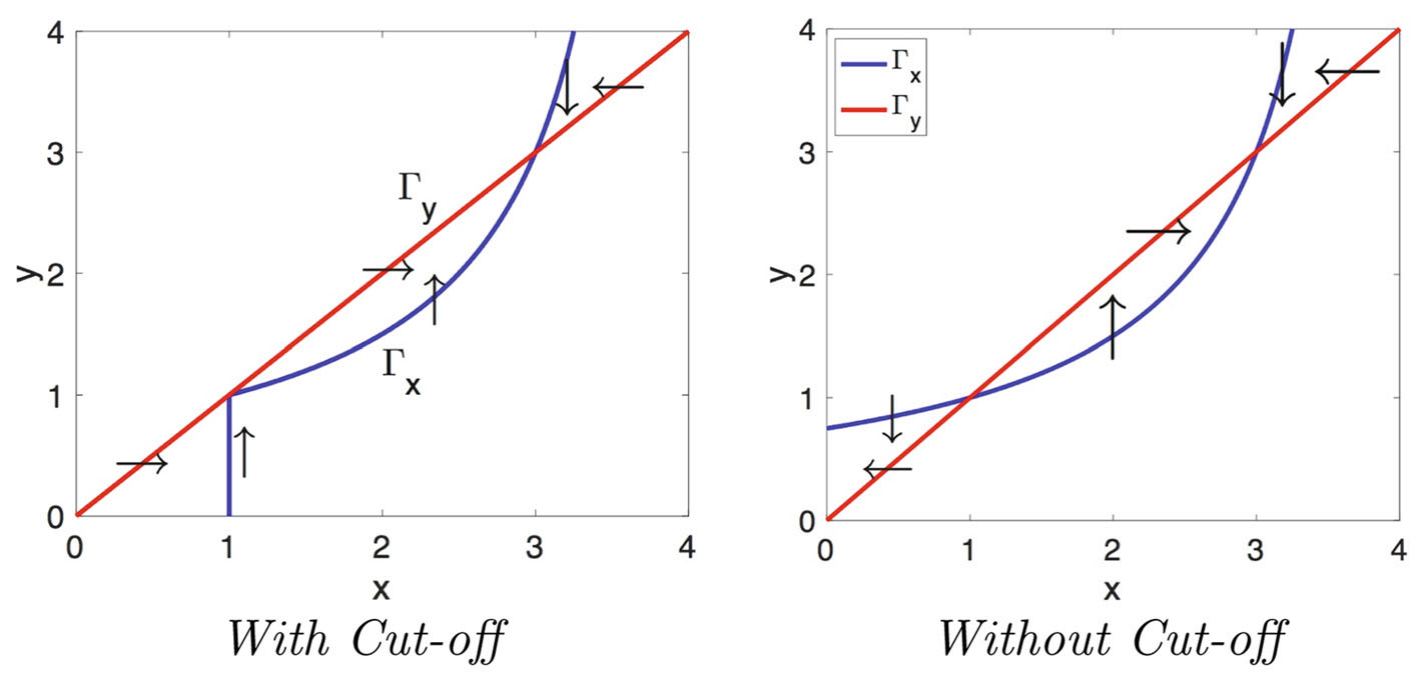
Null clines in the phase plane spanned by the dimensionless concentrations of PRL in lactographs (*x*) and in plasma (*y*) for $\gamma =1$ and $\beta =3$ with Cut-off (*Left*) and without Cut-off (*Right*). The *arrows* indicate the direction of the velocity field **q**: *vertical* on $\Gamma _x$ and *horizontal* on $\Gamma _y$. Note that below the line $y=1$ the direction of the velocity vector **q** has changed: With cut-off it points to the *right* and without cut-off it points to the *left*

Of course, by definition, both *x* and *y* cannot be negative and therefore realistic orbits should lie in the first quadrant. In order to ensure that $x>0$ and $y>0$, Stevens et al. cut off the positive feedback as soon as $R<R_0$ or $y < 1$. This affects the null cline $\Gamma _x$ below the line $y=1$, i.e. in the region $\{(x,y): y<1\}$, as shown in Figure 15. The cut-off makes $\Gamma _x$ vertical below $y=1$. Since the velocity vector on $\Gamma _x$ is also vertical, this section of $\Gamma _x$ is *invariant* and orbits cannot cross it.

Thus, the orbits are unaffected as long as they stay above the line $y=1$. However, when they dip below this line they change direction, move to the right and generally are captured in the triangular region between the two null clines $\Gamma _x$ and $\Gamma _y$ and the *x*-axis. In this region **q** points *up* and *to the right* and orbits can readily be shown to converge to the baseline $(x_0,y_0)=(1,1)$ as time tends to infinity. Thus

59 $$\begin{aligned} x(\tau ) \nearrow 1 \quad {\text {and}} \quad y(\tau ) \nearrow 1 \qquad {\text {as}} \quad \tau \rightarrow \infty \end{aligned}$$

**Fig. 16:**
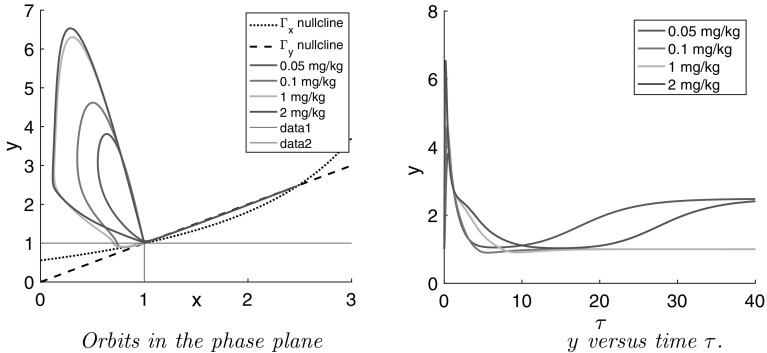
Simulations of the positive feedback model *with cut-off* in the phase plane spanned by the dimensionless concentrations of PRL in lactographs (*x*) and in plasma (*y*) (*Left*) and dimensionless PRL in plasma versus dimensionless time ($\tau$) (*Right*) for the same doses *D* of risperidone as in Fig. 13: *D* = 0.05, 0.1, 1 and 2 mg/kg. The drug PK is simulated using the PK model for *iv* dose of risperidone [34] (cf. Appendix A)

This is what we see demonstrated in the simulations of Fig. 16.

As we have shown, thanks to the cut-off of the positive feedback, we now observe two types of large time behaviour:

(i) Orbits converging to the baseline $(x_0,y_0)$ and
(ii) Orbits converging towards the steady state created by the positive feedback. In both cases, orbits approach their limits from below and from the left. The fact that orbits pass through a neighbourhood of (1,1) makes for very sensitive dependence on the drug dose.

### Conclusion

The mathematical analysis of the prolactin model with positive feedback has explained the unexpected behaviour of orbits and in particular their sensitivity to the drug dose. However, in the process it has done much more and given us great insight in the properties of the model. Thus, it has revealed the existence of two constant steady states, even in the absence of any drug-driven stimulation, i.e., two baselines of which one corresponds to the old baseline $(P_0,R_0)$. Only if $\beta <\gamma$, i.e., if $E_{\mathrm{max}}<(EC_{50}\cdot R_0)$, is the old baseline stable.

## Overall conclusions

We have highlighted the impact of mathematical pharmacology in four case studies derived from previously published data and analyses ([5–7] and [8]). In case study 1, we derive relations between quantities involved in TMDD systems at steady-state, such as target versus ligand and ligand-target complex versus ligand. These relationships give rise to a new expression of *Potency*, $L_{50}$. This potency parameter is a conglomerate of binding affinity ($k_{\mathrm{on}}$ & $k_{\mathrm{off}}$), target turnover ($k_{\mathrm{deg}}$), and ligand-target complex removal ($k_{e(RL)}$). Its applications will range from descriptions of in vitro and in vivo correlations to assessment of determinants of pharmacologically efficacious concentrations.

The second case study involves locomotor activity and demonstrates how mathematical analysis, when combined with pattern recognition, can serve as a refined instrument for extracting qualitative as well as quantitive properties out of data sets, such as model structure, rate constants and dose dependence. Thus, *Visual Inspection* yields valuable parameter estimates which can be used in statistical data analysis for further refinement.

The third case study serves as a warning against the dangers of using multiple rate binding models for extrapolating beyond the experimental time range. This study emphasises the need for long-term data for making small, but in the long term significant corrections to exposure or environmental conditions.

Deeper mathematical study may be required to uncover mysteries in the dynamics of PK-PD systems, such as large computational times or unexpected behaviour. The fourth case study focusses on the latter: a semi-mechanistic pool-precursor model for the dynamics of prolactin is found to exhibit—what appears to be—random dependence on drug dose. Small changes of the dose are seen to cause step changes in terminal behaviour. Thus, mathematical analysis exposes and makes explicit weaknesses of the model.

The take-home messages from this Communication are that mathematical and analytical techniques serve their purpose when we need to chisel out new structures (Case study #1), quantify patterns (Case study #2), make what-if? predictions (Case study #3) and diagnose models with hidden pathologies (Case study #4).

## Appendix

### PK used by Kozielska et al [34]

Kozielsky et al used a simple two-compartment model involving a central and a peripheral compartment in which the drug-amounts were denoted by, respectively, $A_c$ and $A_p$. The drug entered the central compartment from a depot which was supplied by an *iv* bolus dose.

60 $$\begin{aligned} \left\{ \begin{array}{ll} \frac{dA_d}{dt} &{}= - k_a A_d \\ \frac{dA_c}{dt} &{}= k_a A_d - \frac{CL}{V_c}A_c - \frac{CL_d}{V_c}A_c + \frac{CL_d}{V_p}A_p \\ \frac{dA_p}{dt} &{}= \frac{CL_d}{V_c}A_c - \frac{CL_d}{V_p}A_p \\ \end{array}\right. \end{aligned}$$

where $k_a$ denotes the first-order rate with which drug is transported from the depot into the plasma compartment *CL* the clearance from the plasma compartment and $CL_d$ the distributional clearance between the plasma- and the peripheral compartment. The volumes of the plasma- and the peripheral compartment are denoted by $V_c$ and $V_p$, respectively. The PK parameter values used by Kozielska et al are given in Table 5.

**Table 5:** PK parameters used by Kozielska et al [34]

Parameter	Estimate	Units	Description
$k_a$	2.84	L hr$^{-1}$	Influx rate from depot
*CL*	1.62	L hr$^{-1}\cdot$kg$^{-1}$	Clearance
$V_c$	1.29	L kg$^{-1}$	Volume central compartment
$CL_d$	0.0882	L hr$^{-1}\cdot$kg$^{-1}$	Distributional clearance
$V_p$	1.29	L kg$^{-1}$	Volume peripheral compartment

## Footnotes

1
*A problem is well posed if: (i) it has a solution, (ii) the solution is unique, (iii) the solution depends smoothly on parameters and initial data*

2
$T=O(N)$ means that *T* is of the same order of magnitude as *N*.
